# Failure mode and effect analysis for linear accelerator‐based paraspinal stereotactic body radiotherapy

**DOI:** 10.1002/acm2.13455

**Published:** 2021-10-28

**Authors:** Sangkyu Lee, Dale Michael Lovelock, Alex Kowalski, Kate Chapman, Robert Foley, Mary Gil, Gerri Pastrana, Daniel S. Higginson, Yoshiya Yamada, Lei Zhang, James Mechalakos, Ellen Yorke

**Affiliations:** ^1^ Department of Medical Physics Memorial Sloan Kettering Cancer Center New York New York USA; ^2^ Department of Radiation Oncology Memorial Sloan Kettering Cancer Center New York New York USA

**Keywords:** failure modes and effects analysis, quality assurance, root cause analysis, spinal metastasis, stereotactic body radiotherapy

## Abstract

**Introduction:**

Paraspinal stereotactic body radiotherapy (SBRT) involves risks of severe complications. We evaluated the safety of the paraspinal SBRT program in a large academic hospital by applying failure modes and effects analysis.

**Methods:**

The analysis was conducted by a multidisciplinary committee (two therapists, one dosimetrist, four physicists, and two radiation oncologists). The paraspinal SBRT workflow was segmented into four phases (simulation, treatment planning, delivery, and machine quality assurance (QA)). Each phase was further divided into a sequence of sub‐processes. Potential failure modes (PFM) were identified from each subprocess and scored in terms of the frequency of occurrence, severity and detectability, and a risk priority number (RPN). High‐risk PFMs were identified based on RPN and were studied for root causes using fault tree analysis.

**Results:**

Our paraspinal SBRT process was characterized by eight simulations, 11 treatment planning, nine delivery, and two machine QA sub‐processes. There were 18, 29, 19, and eight PFMs identified from simulation, planning, treatment, and machine QA, respectively. The median RPN of the PFMs was 62.9 for simulation, 68.3 for planning, 52.9 for delivery, and 22.0 for machine QA. The three PFMs with the highest RPN were: previous radiotherapy outside the institution is not accurately evaluated (RPN: 293.3), incorrect registration between diagnostic magnetic resonance imaging and simulation computed tomography causing incorrect contours (273.0), and undetected patient movement before ExacTrac baseline (217.8). Remedies to the high RPN failures were implemented, including staff education, standardized magnetic resonance imaging acquisition parameters, and an image fusion process, and additional QA on beam steering.

**Conclusions:**

A paraspinal SBRT workflow in a large clinic was evaluated using a multidisciplinary and systematic risk analysis, which led to feasible solutions to key root causes. Treatment planning was a major source of PFMs that systematically affect the safety and quality of treatments. Accurate evaluation of external treatment records remains a challenge.

## 1 INTRODUCTION

Stereotactic body radiotherapy (SBRT) for treating paraspinal metastasis has been shown to be a highly effective palliative option.^1^, ^2^ SBRT is characterized by irradiation of a relatively small target with a high dose per fraction and few fractions to achieve a higher biological equivalent dose to the tumor. Application of SBRT to spine metastasis, however, poses a number of challenges due to the nature of SBRT and anatomical characteristics.

The challenges in paraspinal SBRT include prescription, treatment planning, and delivery. Intricate balance has to be achieved in the planning stage between local control and dose to the organs at risk (OARs) in proximity to the target volume (TV), such as the esophagus or the spinal cord. Compromise of target coverage, while maintaining an adequate level of local control,^3^ is often inevitable to minimize the risk of radiation myelopathy.^4^ For this reason, an accurate definition of the spinal cord in the simulation computed tomography (CT) is critical. Additional procedures, such as myelogram^5^ or magnetic resonance imaging (MRI),^6^ must be conducted for better visualization of the spinal cord. The consequences of delivery error are especially dire for paraspinal SBRT due to the small number of fractions and the steep dose gradient necessary to treat as much tumor as possible while sparing the cord. Therefore, it is important to minimize setup error and intra‐fractional motion, as small displacements from the planning position might cause a sharp increase in spinal cord dose. Τhis necessitates robust immobilization and an intra‐fractional motion monitoring process that can visualize internal anatomy. Moreover, the linear accelerators (linac) that deliver SBRT are subject to dedicated quality assurance (QA) in order to meet higher standards for dosimetric and geometric accuracy of the delivery.^7^ All of these additional requirements and resources increase the complexity of the patient care workflow, which may increase the risk of failures and near‐misses.^8^

Failure modes and effects analysis (FMEA) is a methodology that systematically analyzes a complex process to identify and evaluate the potential failures that might arise. FMEA can be followed by a fault tree analysis (FTA) to identify the root causes of the identified failures and design solutions that can address the causes.^9^ FMEA was described in the context of radiation oncology workflows by the American Association of Physicists in Medicine Task Group (TG) 100.^9^ It is one of the methods suggested by the American Society for Radiation Oncology for nurturing a strong safety culture.^10^ The FMEA framework has been previously applied to various radiotherapy processes, including intensity‐modulated radiotherapy (IMRT),^9^ brachytherapy,^11^ Cyberknife SBRT,^12^ and liver SBRT.^13^

This work applied the FMEA framework to an institutional paraspinal SBRT program to identify the vulnerabilities in the patient care workflow from simulation to delivery. Furthermore, the work demonstrates how the institution converted the key results from the FMEA into actionable changes to the paraspinal SBRT to enhance its safety.

## 2 MATERIALS AND METHODS

### 2.1 Institutional paraspinal SBRT program

The radiation oncology clinic consists of 33 radiation oncologists, 60 therapists, 40 medical physicists, and 28 dosimetrists (satellite sites were not considered for this study). The spine SBRT program treats approximately 15 metastasis cases per week. Treatment volumes, typically 1–3 vertebral bodies with or without spinous or transverse processes, are currently prescribed with 24 Gy in 1 fraction, 24–30 Gy in three fractions, or 20–40 Gy in five fractions. We prefer that a myelogram be performed as part of the simulation CT scan in order to most accurately contour the spinal cord with the patient immobilized in the treatment position. If a patient is unable to get a myelogram, a T2 weighted diagnostic MRI is acquired and fused to the simulation CT to define the spinal cord. The MRI‐defined spinal cord is expanded by a 1 mm margin in order to take into account the fusion uncertainty. The contours for TVs and certain OARs (spinal cord, cauda, bowel, brachial plexus, stomach, and esophagus) are drawn by the radiation oncologists who are credentialed in spine SBRT per institutional guidelines and peer‐reviewed in weekly volume rounds prior to treatment planning. Treatment planning is performed by a credentialled dosimetrist using the IMRT technique with 7–9 posterior/posterior‐oblique 6 MV flattening filter‐free (FFF) fields. If applicable, the dosimetrist analyzes prior radiotherapy to estimate cumulative dose to OARs and notifies the physician of the estimates via a special physics consult. If the previous treatment was delivered at a different institution, the physician requests the treatment records from the institution. These external treatment records, often with a wide variety of formats and details, are interpreted to the best ability by a dosimetrist for prior OAR dose estimation. If the cumulative dose exceeds certain constraints or satisfies other criteria, a dosimetrist triggers a peer review by another physician, which needs to be completed before delivery. Eclipse and ARIA (Varian Associates, Palo Alto, CA, USA) are used as the treatment planning and management systems, respectively. Patient‐specific plan QA is performed by an in‐house program to confirm that the plan control points that are loaded into the machine are identical to the treatment at the time it received its physics check. All single fraction cases undergo additional patient‐specific measurements with the electronic portal imaging device prior to treatment. Treatments are delivered using a Varian (Varian Associates) TrueBeam linac equipped with a high‐definition 120 (HD120) multi‐leaf collimator. The ExacTrac (BrainLab, Munich, Germany) system is used for monitoring intra‐fractional motion. The quality assurance of the machines and ancillary systems is compliant with the American Association of Physicists in Medicine TG‐142^14^ and Medical Physics Practice Guidelines‐9a^15^ guidelines for SBRT machines. A patient is set up in the treatment position based on registration between a daily cone‐beam CT (CBCT) and the planning CT as performed by a therapist. Then, a pair of ExacTrac images is taken to serve as a baseline for the intra‐fractional motion monitoring. The treatment begins once the image matching is approved by a physician and physics. The position of the vertebral target is monitored using the ExacTrac images that are taken when the gantry is within 10 degrees of the three angles (90°, 180°, 270°). Shifts in target position are calculated by proprietary software based on automatic registration between the baseline and intra‐fractional images, and beam delivery is manually interrupted if the magnitude of the shift exceeds 2 mm.

### 2.2 Risk analysis methodology

Our paraspinal SBRT risk analysis is made up of three sections: 1) Construction of a process map, 2) FMEA, and 3) FTA. Details of each section will be provided below. The risk analysis was conducted by a committee of four physicists, one dosimetrist, two therapists, and two radiation oncologists. All the members of the committee contributed to the sections of the FMEA and the FTA that suit their respective expertise. The project was conducted over 9 months. Most of the communication took place via emails, one‐on‐one phone, or office conversations. Occasional subgroup meetings (e.g., therapists/oncologists) were organized by three physicists who spearheaded the project. The subgroup members spent approximately 3 h, including the survey, informal meetings, and email communications (10 min per email was assumed) while the three organizing physicists spent approximately 10 h to bring the project to the point of implementing the action items described in the Results section.

#### 2.2.1 Process mapping

A process map is a visual representation of a process, illustrating the flow of the specific tasks, or sub‐processes, that are conducted to achieve the objectives of the process.^9^ In this study, the treatment process for patients undergoing paraspinal SBRT within radiation oncology, from a CT simulation appointment to the completion of treatment delivery, was divided into three phases: simulation, treatment planning, treatment delivery. In addition, a process for linear accelerator QA was added due to its importance to the quality of SBRT delivery. A process map was built for each of the four phases. The sub‐processes were organized in chronological order, with the exception of the machine QA, where each sub‐process refers to a test of a certain aspect of machine performance.

#### 2.2.2 Failure modes and effects analysis

The FMEA begins by identifying potential failure modes (PFMs)– any failures or mistakes that can happen – within each sub‐process of the process map from the previous section. The identified PFMs were then evaluated in terms of the following three metrics: 1) Occurrence: how often can the PFM occur?, 2) Severity: how severe is the consequence of the PFM if it reaches the patient undetected?, and 3) Detectability: how likely is it that the PFM reaches a patient undetected? The scoring criteria by Ford et al.^16^ were used (Table 1). Each metric was scored by the committee members for whom it was within their area of expertise on a 1–10 scale, with 10 indicating the highest risk. Average of the scores from multiple committee members were taken for each of the Occurrence/ Severity/Detectability metrics. Then, a product of these three average metrics was taken to obtain the risk priority number (RPN). The three PFMs with the highest RPNs were prioritized for further analysis.

**TABLE 1:** The scoring criteria for evaluating potential failure modes. Reproduced from Ford et al.^16^ with permission

**Score**	**Occurrence**	**Severity**	**Detectability**
1	Less than every 5 years	No effect	
2	Every 2–5 years	Dose change of 5%	Very easy to detect
3	Once a year		
4	Several times a year	Minimal delay in care	Easy to detect
5	Once a month		
6	Several times a month	Allergic reaction, moderate delay in care	Mildly difficult to detect
7	Once a week		
8	Several times a week	Dose change of 20%, reportable	
9	Once a day		
10	Several times a day	Patient dies	Impossible to detect

#### 2.2.3 Fault tree analysis

First, all the possible causes that can directly contribute to the PFM of interest were identified. Then, the lower‐level events that can result in each of these causes were identified. In this fashion, the causes were back‐propagated in a tree‐like structure, until reaching the root causes that fall outside the control of the department.^9^ Last, quality management (QM) measures were designed in a way that they can block the propagation of the causes to a downstream cause or a failure.

## 3 RESULTS

**FIGURE 1:**
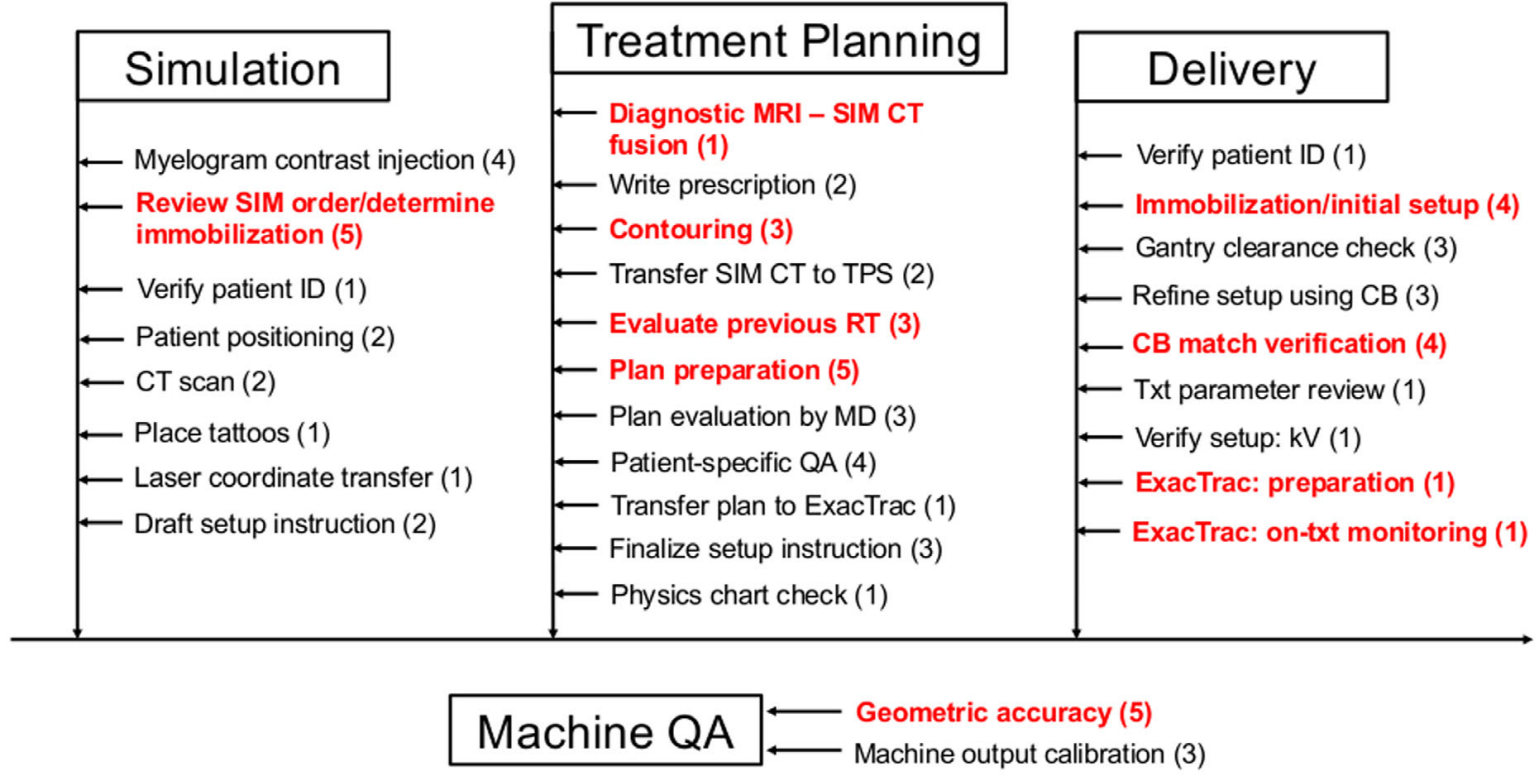
A process map for paraspinal stereotactic body radiotherapy. The numbers in parentheses indicate the number of potential failure modes (PFM) associated with each sub‐process. The sub‐processes highlighted in red indicate the presence of high‐risk PFMs, defined as the upper quartile of the RPN distribution within the corresponding phase. SIM: simulation, CT: computed tomography, QA: quality assurance, CB: cone‐beam CT, txt: treatment

The constructed process maps for simulation, treatment planning, delivery, and machine QA phases are shown in Figure 1. The process was segmented into eight subprocesses in simulation, 11 subprocesses in treatment planning, nine subprocesses in delivery, and two subprocesses in machine QA. A total of 73 PFMs were identified: the number of PFMs identified in each section was 18, 28, 19, and eight for simulation, treatment planning, delivery, and machine QA, respectively. A full list of PFMs and FMEA scores is shown in the Supporting Information 1.

**FIGURE 2:**
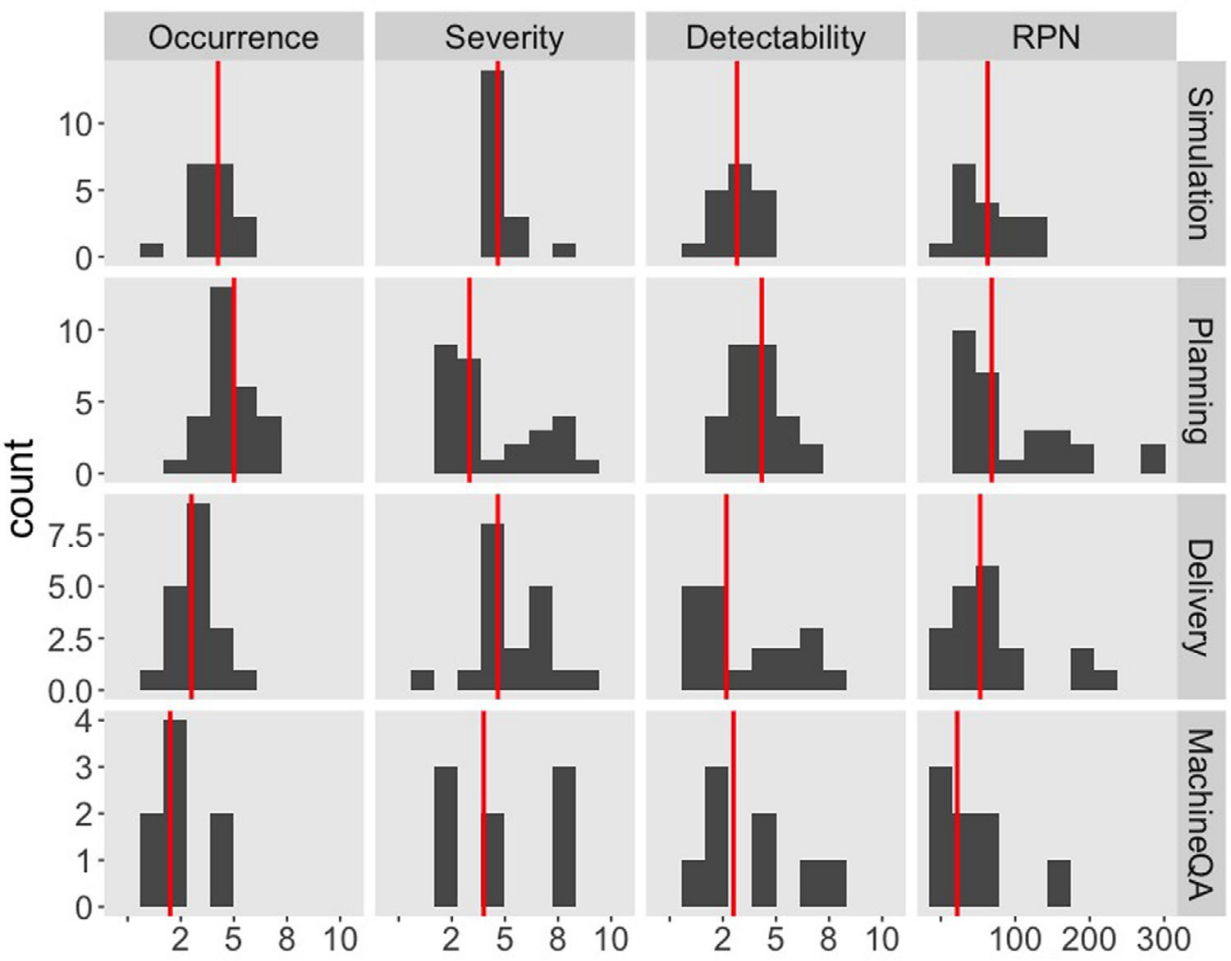
Histograms of failure modes and effects analysis (FMEA) scores for the 73 potential failure modes from machine quality assurance (QA), planning, simulation, and delivery subsections. Red vertical lines represent a median value

Distributions of the average FMEA metrics scored by the committee members are shown in Figure 2. On the whole, the PFMs from treatment planning showed more frequent occurrence (median O: 5.0) and were harder to detect (median D: 4.3) than the other subdivisions. As a result, the median RPN was the highest (68.3) from the treatment planning process. Median severity was the highest for simulation (4.7) and delivery (4.7). The distribution of severity scores for treatment planning and detectability scores for delivery appeared to be bimodal. There were a small number of PFMs scoring much higher than the level that a median value indicates.

**FIGURE 3:**
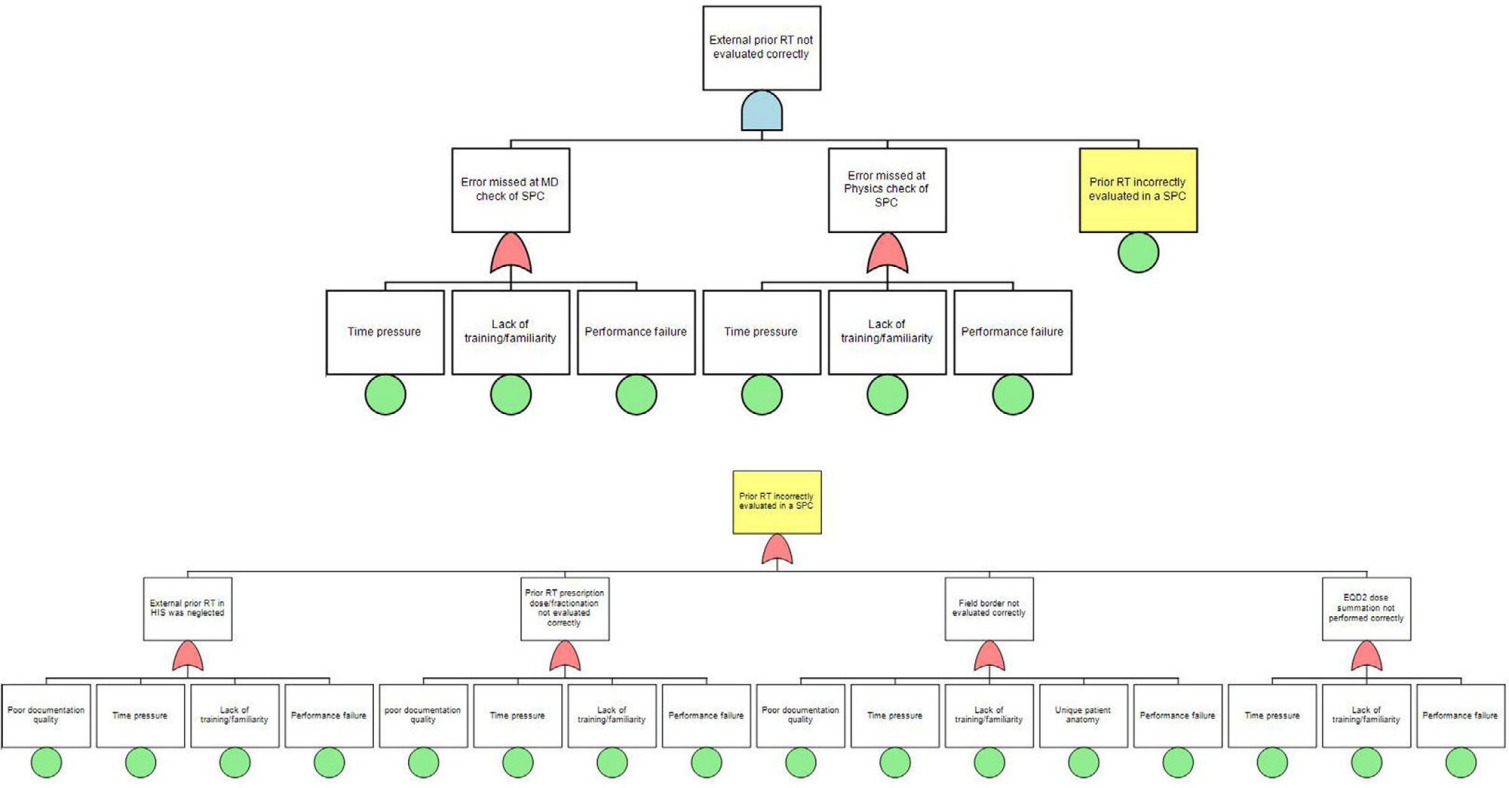
A fault tree for the potential failure mode “external prior radiotherapy not evaluated correctly”. Failure of quality assurance or control propagates upstream through AND gates, while the other potential failures propagate through OR gates. The node highlighted in yellow is expanded to another failure tree (bottom). EQD2: equivalent dose in 2 Gy fraction, HIS: health information system, SPC: special physics consult

**FIGURE 4:**
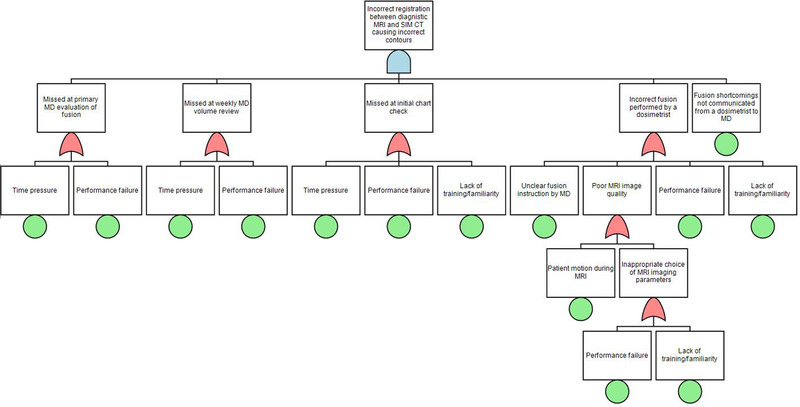
A fault tree for the potential failure mode “incorrect registration between diagnostic magnetic resonance imaging (MRI) and simulation computed tomography (CT) causing incorrect contours”. SIM: simulation

**FIGURE 5:**
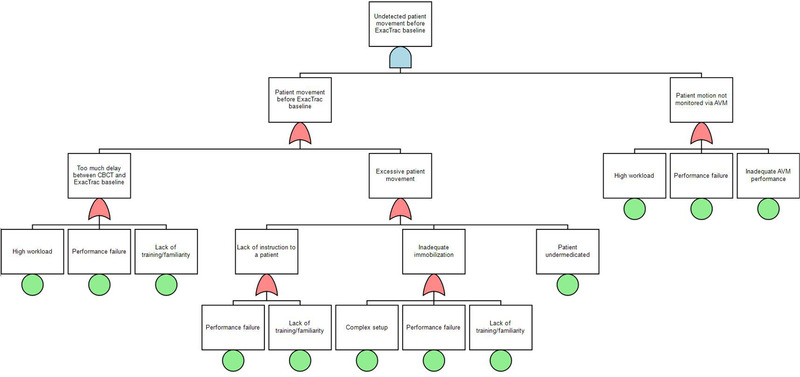
A fault tree for the potential failure mode “undetected patient movement before ExacTrac baseline”. DRR: Digitally reconstructed radiograph, CBCT: cone‐beam CT, AVM: audiovisual monitor

The five PFMs with the highest RPN are shown in Table 2. The FTAs were performed on the three PFMs with RPN > 200. These PFMs originated respectively from the sub‐processes evaluate previous RT, Diagnostic MRI–CT SIM fusion, and ExacTrac preparation. The fault tree for the PFM previous treatment outside the institution is not/cannot be accurately evaluated is shown in Figure 3. The identified root causes, shown as terminal nodes in the tree, were time pressure, lack of training/familiarity, performance failure (any mistakes made randomly by an operator not caused by time pressure or lack of training), poor documentation, and challenging patient anatomy. Figure 4 illustrates a fault tree for the PFM incorrect registration between diagnostic MRI and simulation CT causing incorrect contours–time pressure, lack of training/familiarity, and performance failure was again identified as root causes. The other causes were poor communication between a physician and a dosimetrist, and patient motion during MRI. The fault tree for the third PFM patient movement before the ExacTrac baseline affecting treatment undetected is illustrated in Figure 5. The identified root causes were lack of training/familiarity, performance failure, high workload, and inadequate audiovisual monitoring.

**TABLE 2:** Five potential failure modes with the highest risk probability number (RPN). O: Occurrence, S: Severity, D: Detectability. RT: Radiotherapy, SIM: Simulation, CB: Cone beam

**Phase**	**Sub‐process**	**Potential failure modes**	**O**	**S**	**D**	**RPN**
Treatment planning	Evaluate previous RT	Previous treatment outside the institution is not/cannot be accurately evaluated by a dosimetrist	5.5	8.0	6.7	293.3
Treatment planning	Diagnostic MRI–CT SIM fusion	For patients with the cord defined by MRI, poor fusion causing wrong cord location	7.0	6.5	6.0	273.0
Delivery	ExacTrac: preparation	Undetected patient movement before ExacTrac baseline	4.0	6.7	8.2	217.8
Treatment planning	Plan preparation	Plan violates a normal tissue limit and no peer review is done	5.0	7.3	5.0	183.3
Delivery	CB match verification	Patient moves in between cone‐beam acquisition and physician's approval	4.0	6.7	6.8	182.2

The findings from this study led to several actions and changes to the workflow that were attempted to address the common root causes of the three PFMs.

1. Prior RT evaluation: We opened educational and discussion sessions on prior RT evaluation, which helped educate the staff on accurate evaluation and effective communication between dosimetrists, physicists, and physicians. These sessions were recorded for availability to future staff and to refresh the understanding of current staff.
2. MRI–CT fusion: We developed a standard MRI acquisition protocol to be used at the diagnostic MRI scanner, which reduces the variability of MR image quality and field of view.
3. In addition, a dedicated workflow was developed in the fusion software to streamline the paraspinal fusion process, and more importantly, to assist identification of the correct vertebrae to be treated. Also, an educational session on the use of the new workflow was provided and recorded.
4. The use of the ExacTrac: This study prompted therapists to review their practice on ExacTrac use and train the staff so that the delay in baseline ExacTrac image acquisition is minimized. This was also introduced in a revised therapist competency form.

## 4 DISCUSSION

Traditionally, QM efforts in radiation oncology have been fragmented into individual components of the process. For example, quality assurance of linear accelerators is typically conducted under calibration conditions, separated from actual patient care, and thus is limited in predicting the issues that could happen during treatments. In contrast, FMEA provides an integrated approach that evaluates how well these individual components are connected, which components deserve more effort, and the weighting of resources towards improving safety. Findings from this FMEA, applied to the institutional paraspinal SBRT program, highlighted the high‐risk PFMs that arise from suboptimal process inputs or human factors that are not accounted for by either routine QA such as patient‐specific measurements, physics chart check, and linac's QA, or even the end‐to‐end tests. This discovery was made possible by opening up lines of communications between department subgroups (therapists, physicians, physicists) and encouraging each group to reevaluate its workflow to account for its impact on the other groups and on the overall treatment quality, which is not a trivial endeavor in a large institution.

The PFM with the highest RPN pertains to incorrect evaluation of prior RT from external institutions. The high RPN was caused by very high severity (8), high detectability (6.7), and moderate occurrence (5.5). Evaluation of previous treatments is becoming more common as the survival rate for metastatic diseases improves.^17^ In our institution, different planning objectives and constraints are given to the subset of the OARs that are included in previous treatment fields. Incorrect outcomes of this process can lead to excessive OAR dose if the prior dose is underestimated, or loss of local control to the contrary. Analysis of prior RT is a manual process, and its accuracy depends heavily on dosimetrists’ experience and the quality of treatment records. The new training program was designed to address common problems and techniques to best use available prior treatment documentation. However, the varying quality of treatment records from external institutions contributes to inaccuracies that are difficult to detect at physician's plan review or conventional physics chart check. Quality control (QC) of these external records is challenging. A potential remedy is to facilitate the exchange and standardization of radiotherapy records across institutions. However, this solution reaches beyond institutional efforts, and collaboration within the radiation oncology community, such as the survivorship passport for pediatric patients in Europe,^18^ would be necessary.

**FIGURE 6:**
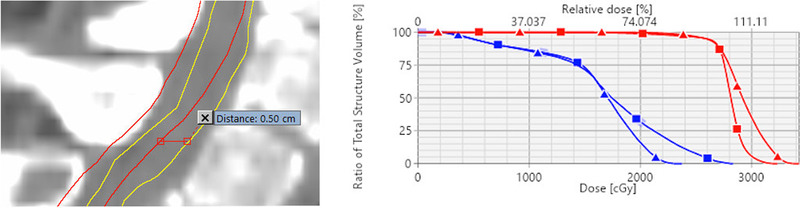
A case of the incorrect spinal cord contour and its influence on the optimized plan. Left: the spinal cord contour was initially drawn based on incorrect fusion (red) and later corrected to the correct position, which was 0.5 cm anterior (yellow). Right: Cumulative dose‐volume histograms for a planning target volume (red) and the spinal cord (blue) from two treatment plans that were separately optimized to the incorrect (square) and correct (triangle) cord contours

Another significant source of failure in the treatment planning phase is a fusion between diagnostic MRI and simulation CT for localizing the spinal cord for patients who do not have a myelogram. Currently, our workflow requires that such fusions be reviewed by the prescribing physician and the physicist performing the chart review. In one case where the poor quality of fusion was detected by a physician, the maximum dose to cord could have increased by 4.6 Gy and planning target volume (PTV) mean dose could have been sacrificed by 1.2 Gy if the cord contour had been drawn based on a wrong fusion (Figure 6). The spine MRI–CT fusion relies on dosimetrists’ experience, and it is further complicated by challenging anatomy and sub‐optimal image quality. We introduced standardized MR image acquisition parameters to be used by diagnostic MRIs acquired in response to a Radiation Oncology request and a streamlined workflow in the fusion software to reduce fusion variability and enhance the QC of imaging data for contouring. The FTA pointed out that adequate and timely communication between the dosimetrist and physician is essential, as the fusion uncertainty could eventually affect plan quality. The new training on image fusion emphasized an appropriate use of the special physics consult to communicate fusion uncertainty. Further actions to address this PFM could include a more automated image registration algorithm that requires less manual tuning, and MRI‐only treatment planning that obviates the need for the fusion.

The PFM with the highest RPN from the delivery phase pointed to a vulnerability in our Image‐guided radiation therapy procedure: patient motion between the last CBCT acquisition and the acquired baseline ExacTrac image was not detectable. This PFM recorded the highest detectability score amongst all PFMs (8.2). The baseline ExacTrac images cannot be reliably compared to the simulation CT to detect motion, due to concerns with false‐positive indications of movement that can be caused by the appearance of overlying bony structures in the 2D images, imaging artifacts, or imperfect reconstruction of digitally reconstructed radiograph from the simulation CT.^19^ The current policy is to acquire the ExacTrac baseline immediately after a patient is set at a treatment position using CBCT, thereby minimizing the time window between the CBCT and ExacTrac images where patient motion is not captured by the ExacTrac system. However, depending on the complexity of the setup and the level of therapists’ attention, the baseline acquisition could be delayed. This PFM was addressed by revised staff training and credentialling to enforce the policy to minimize the delay. A potential long‐term solution that we have not implemented is to establish a connection between ExacTrac and the linac to enable automatic triggering of ExacTrac image acquisition right after CBCT.

Several departmental efforts to encourage safer paraspinal SBRT treatments preceded this FMEA. For example, a peer review triggering by a dosimetrist for the plans at higher toxicity risk was designed to provide an opportunity for another physician to review the plan before a treatment begins, thereby complementing chart rounds (which often reviews an SBRT plan after most fractions have already been delivered). Another challenge we face in a large clinic is time pressure, which has been noted as a root cause for several PFMs. In response, the department developed an in‐house software suite, known as the expedited and constrained hierarchical optimization^20^ to automate the IMRT optimization process, and paraspinal SBRT was the first application of this technology. Shortened optimization time was expected to reduce time pressure on the dosimetrists to allow them to better focus on safety aspects such as prior RT evaluation. However, the introduction of such automatic tools might result in over‐confidence in automation. During our FMEA investigation, it was suggested that expedited and constrained hierarchical optimization might give the impression that paraspinal SBRT planning can be completed in a short time, even though relevant prior RT still has to be analyzed manually and can affect the planned dose. This gap between the perceived and the actual workload might lead to rushed prior RT evaluation and consequent mistakes. We are seeking a way to evaluate the expected workload for a given case at the time of simulation, which can be used for optimizing the plan processing time and helping dosimetrists to prioritize their work.

It is noteworthy that the PFMs from the simulation phase as a whole showed lower RPN than the planning or delivery phases, which is driven by lower detectability scores (Figure 2). There are two possible reasons: first, QC on simulation is enforced during the treatment planning phase and some serious PFMs can be corrected via re‐simulation. Second, the simulation process is standardized for corresponding anatomical regions (cervical, thoracic, and lumbar/sacral spine) with relatively little inter‐patient variability.

The quantified risk from the machine QA component was relatively low thanks to stringent linac's QA, preventive maintenance, and an interlock system that prevents the errors from propagating. The PFM associated with position steering error on six FFF beams presented with the highest RPN (160.0) of all machine PFMs, due to the difficulty in detecting it during routine QA such as the electronic portal imaging device‐based patient‐specific QA (D = 8.0). Beam steering error results in an increase in the size of the radiation isocenter and thus deviation of the delivered dose distribution from the planned one. However, the beam spot size of the six FFF beams is not checked in the machine's daily self‐check (machine performance check process).^21^ Due to the independence of the linac's steering mechanism for each energy,^22^ an independent check on beam geometry for FFF beams would improve the detectability of this PFM. In response to this PFM, an isocenter size measurement for six FFF beams, based on the Winston‐Lutz analysis, was introduced as part of linac's annual QA.

Several differences are notable between this study and earlier FMEAs in related areas. Huq et al.^9^ highlighted in the TG‐100 report that incorrect contouring of TVs and OARs were the PFMs with the highest RPN. In our analysis, the corresponding PFMs were also present but were ranked relatively low (Supporting Information 1). One explanation for this difference is that the PFMs in TG‐100 were scored under the assumption that there are no QA/QC steps in place, which was not the case in this study. Therefore, the difference in quantified risk between the two studies elucidates the effectiveness of the QM efforts already in place at our institution. Specifically, a weekly contouring review session for physicians treating paraspinal tumors was initiated in 2019, and it was instrumental in reducing the incidence of contouring errors and allowing more standardization of contouring practices. Moreover, a credentialing process for treatment planners and physicians with a focus on the correct interpretation of images and contours was instituted further bolstering image registration and contour review. Veronese et al.^12^ conducted FMEA on Cyberknife‐based liver and spine SBRT and reported the highest RPN as being the failures in delivering different treatment parameters from an approved treatment plan. Such a risk was mitigated in our institution by the in‐house and vendor‐provided treatment data integrity verification system, a chart review by therapists prior to beam on, and a standardized treatment technique. In contrast, many of the PFMs with high RPNs in our study were identified in the treatment planning phase. This is also reflected in other studies reporting treatment planning as the most common source of safety incidents,^23^, ^24^ emphasizing the importance of physics chart review.^25^

One of the limitations of this study is due to the semi‐quantitative nature of FMEA. Scoring of risk metrics was subjective and not entirely based on quantitative evidence, and thus could be influenced by a personal bias or the experience level of an individual scorer. In addition, we followed the risk assessment scheme by Ford et al.^16^ instead of TG‐100, because it was practically difficult to perform risk evaluation under the hypothetical assumption of no QM in place. As a result, our results are to some degree tied to the practices in this institution. Despite these limitations, the results from this study could be translated to other institutions with a similar design of workflow and QM measures. Moreover, in the wake of the recently shown benefit of SBRT over conventional RT for spine metastasis,^26^ we hope that our results will serve as a useful resource for setting up new paraspinal SBRT programs.

## 5 CONCLUSIONS

The safety of the institutional paraspinal SBRT program was evaluated using a standard FMEA approach. This analysis highlighted external prior RT evaluation, CT to MRI fusion, and the use of ExacTrac to prevent intrafractional motion as the PFMs that deserve more attention and resources for improving our current practice. Fault tree analysis was performed on these PFMs to inform the complementary QM measures. These measures, all feasible to implement, involved staff education on prior RT evaluation, MRI–CT fusion and appropriate timing of ExacTrac image acquisition, additional QC on MRI acquisition and streamlined MRI–CT fusion workflow, and additional linac's QA on six FFF treatment beams. However, QC of external prior RT records and exploitation of treatment planning automation tools to ensure safety remains a challenge.

## CONFLICT OF INTEREST

The authors declare that there is no conflict of interest.

## AUTHOR CONTRIBUTIONS

Sangkyu Lee contributed to conceptualization, methodology, risk scoring, data analysis, writing, reviewing, and editing. Ellen Yorke and Michael Lovelock contributed to conceptualization, methodology, risk scoring, data analysis, reviewing, and editing. Alex Kowalski, Kate Chapman, Robert Foley, and Mary Gil contributed to risk scoring, reviewing, and editing. Daniel S. Higginson and Yoshiya Yamada contributed to conceptualization, reviewing, and editing. Gerri Pastrana, Lei Zhang, and James Mechalakos contributed to data analysis, reviewing, and editing.

## Supporting information

Supporting information
